# Bead Arrays for Antibody and Complement Profiling Reveal Joint Contribution of Antibody Isotypes to C3 Deposition

**DOI:** 10.1371/journal.pone.0096403

**Published:** 2014-05-05

**Authors:** Burcu Ayoglu, Eszter Szarka, Krisztina Huber, Anita Orosz, Fruzsina Babos, Anna Magyar, Ferenc Hudecz, Bernadette Rojkovich, Tamás Gáti, György Nagy, Jochen M. Schwenk, Gabriella Sármay, József Prechl, Peter Nilsson, Krisztián Papp

**Affiliations:** 1 Affinity Proteomics, SciLifeLab, School of Biotechnology, KTH Royal Institute of Technology, Stockholm, Sweden; 2 Department of Immunology, Eötvös Loránd University, Budapest, Hungary; 3 MTA-ELTE Research Group of Peptide Chemistry, Budapest, Hungary; 4 Department of Organic Chemistry, Eötvös Loránd University, Budapest, Hungary; 5 Department of Rheumatology, Polyclinic of the Hospitaller Brothers of St. John of God, Budapest, Hungary; 6 Department of Genetics, Cell and Immunobiology, Semmelweis University, Medical School, Budapest, Hungary; 7 MTA-ELTE Immunology Research Group, Budapest, Hungary; 8 Diagnosticum Ltd., Budapest, Hungary; Oxford University, United Kingdom

## Abstract

The development of antigen arrays has provided researchers with great tools to identify reactivities against self or foreign antigens from body fluids. Yet, these approaches mostly do not address antibody isotypes and their effector functions even though these are key points for a more detailed understanding of disease processes. Here, we present a bead array-based assay for a multiplexed determination of antigen-specific antibody levels in parallel with their properties for complement activation. We measured the deposition of C3 fragments from serum samples to reflect the degree of complement activation via all three complement activation pathways. We utilized the assay on a bead array containing native and citrullinated peptide antigens to investigate the levels of IgG, IgM and IgA autoantibodies along with their complement activating properties in serum samples of 41 rheumatoid arthritis patients and 40 controls. Our analysis revealed significantly higher IgG reactivity against the citrullinated fibrinogen β and filaggrin peptides as well as an IgA reactivity that was exclusive for citrullinated fibrinogen β peptide and C3 deposition in rheumatoid arthritis patients. In addition, we characterized the humoral immune response against the viral EBNA-1 antigen to demonstrate the applicability of this assay beyond autoimmune conditions. We observed that particular buffer compositions were demanded for separate measurement of antibody reactivity and complement activation, as detection of antigen-antibody complexes appeared to be masked due to C3 deposition. We also found that rheumatoid factors of IgM isotype altered C3 deposition and introduced false-positive reactivities against EBNA-1 antigen. In conclusion, the presented bead-based assay setup can be utilized to profile antibody reactivities and immune-complex induced complement activation in a high-throughput manner and could facilitate the understanding and diagnosis of several diseases where complement activation plays role in the pathomechanism.

## Introduction

Antigen array-based methods allow screening for hundreds or thousands of potential targets of antibody reactivities and they are being increasingly utilized to identify novel antibody reactivities in the context of various pathological conditions such as autoimmune diseases [1]–[3]. The focus of such approaches is mostly limited only to determine the targets of antibodies, usually of IgG isotype. Yet, the information on targets of autoantibodies or antibodies towards infectious agents can be further enriched by screening for other antibody isotypes and by studying their effector functions.

Antigen-antibody immune complexes can induce various effector functions including activation of the complement system, yet the degree of antibody-induced complement activation is influenced by various factors such as antibody isotype composition, antibody affinity or glycosylation state [4]. Investigating the effector functions of antigen-antibody complexes can therefore potentially add another valuable dimension for categorizing antigens or classifying clinical samples in any given autoimmune or infectious disease.

The complement system is one of the first protection lines against pathogens. More than 30 proteins form an orchestrated enzyme cascade, which can be activated by classical, lectin or alternative pathways of the complement system. All these three pathways converge at the point of cleavage of the protein C3, the third complement component, followed by activation of the common terminal pathways [5]. Various molecular structures are able to initiate the activation of the complement system, *e.g.* immune complexes (classical pathway); certain carbohydrates (lectin pathway); or several microbes or aggregates of immunoglobulins (alternative pathway) [6]. The resulting complement cleavage products induce multiple immunological effects like opsonization of the antigen, induction of inflammation or lysis of certain pathogens; some of these effects are important not only for protection against invading microbes but also play a role in autoimmune diseases.

Rheumatoid arthritis (RA) is a systemic, inflammatory, autoimmune disease that affects 0.5–1% of the world population [7]. RA is characterized by formation of rheumatoid pannus in synovial membranes, which erode adjacent cartilage and bone, subsequently leading to joint destruction [8], [9]. Rheumatoid factors (RF) – antibodies specific to the Fc part of IgG – can be detected in up to 70–80% of RA patients [10], but the specificity of RFs is not adequate as positive results occur in other autoimmune and infectious diseases and in up to 15% of healthy individuals, too [9], [11]. Therefore, RF performs poorly as a screening test and should not be ordered in patients with minimal or no symptoms. Autoantibodies against citrullinated forms of peptides derived from various proteins are being suggested as promising disease markers for RA [12]. Citrullinated filaggrin was the first recognized protein, which proved to be a good candidate for detection of autoantibodies against anti-citrullinated peptides (ACPAs) [13]. But filaggrin is expressed in epithelial and not in RA-affected articular tissue, so citrullinated filaggrin is not the *in vivo* target but rather represents an *in vitro* cross-reactive antigen of ACPAs [14]. Masson-Bessière *et al.* showed that the main *in vivo* targets of citrullinated filaggrin-specific antibodies are deiminated forms of fibrinogen α and β chains, which are present in rheumatoid synovial membranes [8]. Other citrullinated protein targets in the joints like vimentin, collagen type II and α-enolase have also been previously investigated and some more are waiting for further characterization [12]. ACPA and RF define overlapping populations of RA patients. Joint destruction, comorbidities such as cardiovascular disease and other extra-articular manifestations are all prominent in the subset of patients positive for RF and ACPA [15].

The target of ACPA might be not only an autoantigen but also a viral antigen such as the citrullinated form of Epstein-Barr virus encoded nuclear antigen (EBNA-1), suggesting a possible role of Epstein-Barr virus (EBV) infection in the induction of disease-specific antibodies in RA [16]. EBV is a human γ-herpesvirus that infects more than 90% of the world population. EBV persists lifelong in the human host after the primary infection and is well controlled by the host's immune system, but it can cause a number of malignancies of lymphoid and epithelial origin in immunosuppressed individuals. EBNA-1 is expressed in all EBV infected cells and is the only viral protein regularly detected in all malignancies associated with EBV [17]–[19]. Antibodies against citrullinated peptides derived from EBNA-1 in contrast to the native form can differentiate between RA from non-diseased samples [16].

Assay setups based on suspension bead arrays currently offer a great sample throughput compared to planar array-based assay, allowing a highly multiplex analysis of up to 384 analytes in up to 384 samples per assay run [20]. Despite the presence of studies utilizing planar antigen arrays, there are no established protocols available to implement suspension bead-arrays for studying antigen-specific antibody reactivity and complement activation induced C3 deposition in a single assay. Here we describe development of an antigen bead-array based method for parallel detection of antibody isotypes and measurement of their complement activation properties. Our aim was to determine the optimal conditions and establish a workflow for parallel analysis of antibody reactivity and immune-complex induced complement activation on a suspension bead array platform. We demonstrated the utility of our method in the context of RA by screening serum samples for reactivity against citrullinated and non-citrullinated forms of various RA-related peptides. Furthermore, we tested the viral antigen EBNA-1 specific humoral responses in this serum sample cohort.

## Materials and Methods

### Ethics Statement

Serum samples were collected after written consent with ethical permission of the Scientific Research Ethics Committee of the Medical Scientific Board of Ministry of Human Resources (ETT TUKEB 5257-0/2010-1018EKU 376/PI/010).

### Serum samples

Serum samples were collected from 41 RA patients with established disease and from 40 healthy volunteers. The diagnosis of the disease was established on the basis of the revised ACR/EULAR classification criteria [21]. Sample donor information for RA patients and non-diseased controls is summarized in Table 1. Serum samples were aliquoted within less than 2h after blood collection and were stored at –70°C without being exposed to any freeze-thaw cycle to preserve the complement activating property.

**Table 1:** Demographics of the serum sample donors.

Atribute	RA Group	Control Group
Male/Female	10/31	13/27
Age (year)	56 (21–84)^a^	29 (19–65)
CCP2 Test +/−	38/3	
DAS index	4.2 (0.6–8.2)	
Disease duration (year)	4.5 (0–32)	

a Median and ranges in brackets are shown, where applicable. DAS - disease activity score.

### Peptide synthesis

The N-terminally biotinylated fibrinogen β chain (UniProt: P02675) derived peptides (Biotinyl-SGSG-^60^RPAPPPISGGGYRAR^74^ or Biotinyl-SGSG-^60^ **X**PAPPPISGGGY**X**A**X** ^74^ where X stands for citrulline) [14] were purchased from Mimotopes Ltd. (Australia). The C-terminally biotinylated filaggrin (UniProt: P20930) related peptides (^454^TRGRS^458^-K-LCbiotin-Ttds or ^454^T**X**GRS^458^-K-LCbiotin-Ttds) were synthesized manually by solid phase peptide synthesis according to Fmoc/^t^Bu strategy using biotinyl-6-aminohexanoic acid (LCbiotin) and 4,7,10-trioxa-1,13-tridecanediamino succinic acid linker (Ttds) and biotin [22], [23]. Lysine was added to the C-terminus of the peptide for conjugation of the biotin derivatives to its ε-amino group of the side chain. The crude products were purified by HPLC. The amino acid sequence of the peptides was confirmed by ESI mass spectrometry.

### Proteins and secondary antibodies

Properdin (Calbiochem); human IgG, IgM and IgA (Sigma); EBNA-1 protein (Tebu-Bio) and neutravidin (Thermo Scientific) were coupled to beads and utilized to determine the optimal assay conditions for measurement of complement activation. For detecting the various antibody isotypes, goat anti-human IgM (µ chain specific) F(ab')_2_ –Cy3, goat anti-human IgG (γ chain specific) F(ab')_2_ –PE or goat anti-human IgA (α chain specific) F(ab')_2_ –DyL549 (Jackson ImmunoResearch Laboratories) antibodies were used. Goat anti-human complement C3 specific F(ab')_2_ antibody (MyBioSource) was labeled with R-Phycoerythrin (Lightning-Link-R-Phycoerythrin Conjugation Kit, Innova Biosciences) according to manufacturer's protocol and was used to detect complement activation driven C3 deposition on beads.

### Generation of suspension bead arrays

Proteins were coupled to carboxylated magnetic beads (MagPlex-C, Luminex Corp.) based on a previously published antigen coupling protocol [2] with minor changes. In brief, 10^6^ beads per bead identity were distributed across 96-well plates (Greiner BioOne), washed and re-suspended in phosphate buffer (0.1 M NaH_2_PO_4_, pH 6.2) using a plate magnet and a plate washer (EL406, Biotek). The carboxyl groups on the surface of the beads were activated by 0.5 mg of 1-ethyl-3(3-dimethylamino-propyl) carbodiimide (Pierce) and 0.5 mg of N-hydroxysuccinimide (Pierce) in 100 µl phosphate buffer. After 20min incubation on a shaker (Grant Bio), beads were washed in MES buffer (0.05 M 2-(N-morpholino)ethanesulfonic acid, pH 5.0). For coupling of the following proteins to beads, 2-fold serial dilutions of the proteins with indicated starting concentrations were prepared in MES buffer: human IgG, IgM, IgA and properdin at 160 µg/ml; recombinant EBNA-1 protein at 80 µg/ml. Beads coupled in protein-free MES buffer were used as negative control. The coupling reaction was allowed to take place for 2h at RT, the beads were then washed 3x in PBS-T (0.05% Tween20 in PBS), re-suspended in 100 µl storage buffer (1% BSA, 0.05% Tween20, ProClin300 in PBS) and stored in plates at 4°C overnight. For efficient coupling of the peptides to the beads, the bead surface was first modified by covalent coupling of neutravidine (250 µg/ml) as described above. Then, 100 µl of each biotinylated peptide solution at 50 µM concentration was added to neutravidin-coated beads. Following an overnight incubation at 4°C, beads were washed 3x in PBS-T and re-suspended in 100 µl storage buffer. The final antigen suspension bead array was prepared by combining equal volumes of each bead identity and the mixture was re-suspended in storage buffer. After adjustment of the final volume to enable the transfer of 5 µl of bead solution per well, the beads were sonicated (Branson Ultrasonic Corp) and stored at 4°C until further use.

### Assays on suspension bead arrays

**Figure 1:**
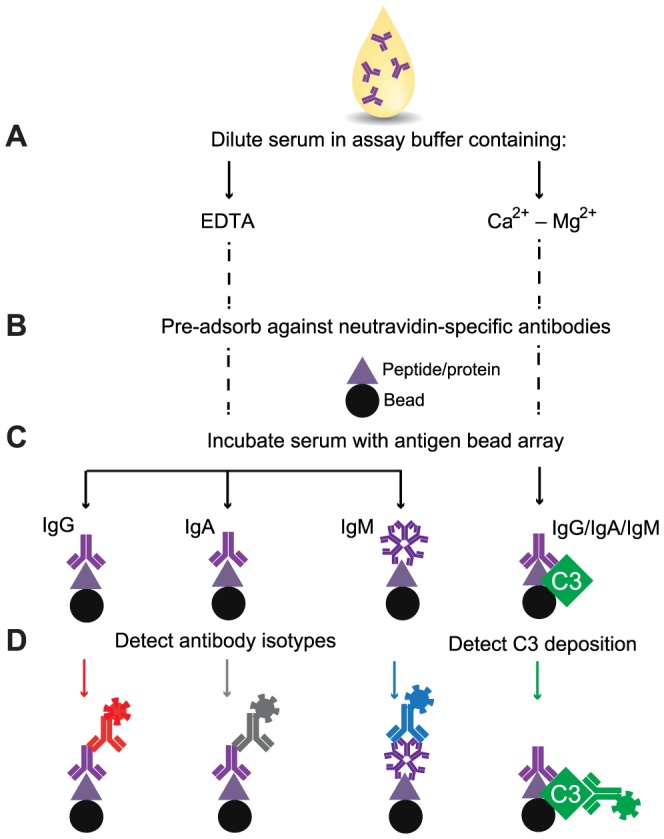
Schematic representation of assay workflow. **A**) Serum samples are diluted 1∶10, either in a Ca^2+^-Mg^2+^ containing assay buffer for detection of complement activation, or in an EDTA containing assay buffer for antibody detection. B) The samples are pre-adsorbed against neutravidin-specific antibodies. **C**) A mixture of beads coupled to various antigens is distributed into a 384-well plate and the pre-adsorbed samples are added to the bead array. **D**) Complement activation driven C3 deposition and different antibody isotypes are detected in parallel with fluorescently labeled secondary antibodies dispensed into individual wells of each quadrant of the 384-well plate.

Two types of assay buffers were utilized for the analysis of serum samples. An assay buffer supplemented with Ca^2+^ and Mg^2+^ (2.5 mM Ca^2+^, 0.7 mM Mg^2+^, 5% BSA, ProClin300 in PBS) was used for measurement of deposited C3 fragments, whereas an assay buffer supplemented with EDTA (25 mM EDTA, 5% BSA, ProClin300 in PBS) was used for detection of various antibody isotypes. Serum samples were diluted 1∶10 (v/v) in assay buffer supplemented with 100 µg/ml neutravidin and incubated for 45 min on ice for pre-adsorption against neutravidin-specific antibodies. Using a liquid handler (CyBi-SELMA, CyBio), 50 µl of 1∶10 diluted serum samples were transferred to 384 well plates containing 5 µl bead array per well. Following incubation at 37°C on a shaker (Grant Bio) for 1h, beads were washed 6x with 60 µl PBS-T on a plate washer (EL406, Biotek) and re-suspended in 50 µl of each secondary antibody solution. Anti-human C3–PE was used for detection of complement activation, whereas anti-human IgM–Cy3, anti-human IgG–PE or anti-human IgA–DyL549 antibodies were used for detection of bound antibody isotypes (**Figure 1**). All secondary antibodies were diluted 1∶500 in a buffer consisting of 5% BSA, 0.05% Tween20 in PBS. After incubation with the secondary antibodies for 30 min, the beads were washed 3x with 60 µl PBS-T and re-suspended in 60 µl PBS-T for measurement by a FlexMap3D instrument (Luminex Corp.). At least 50 events per bead identity were counted and binding events were displayed as median fluorescence intensity (MFI) values.

### Data Analysis

Data analysis and visualizations were performed using R [24] and various R packages or Prism 4 (GraphPad Software Inc.). Non-parametric Spearman's correlation and Mann-Whitney test were used, values were considered statistically significant if p-value < 0.05.

## Results

### Effect of serum sample dilution rate on complement activation

**Figure 2:**
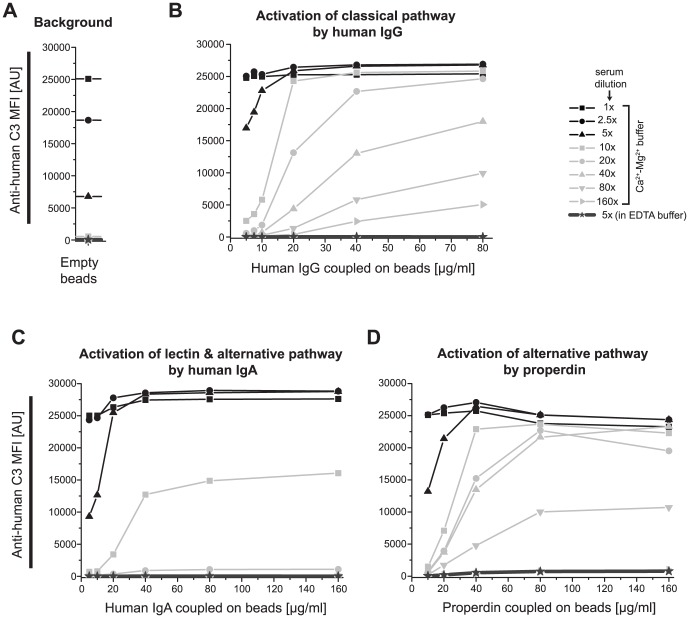
Dependence of complement activation on serum dilution rate. For measuring the background, classical, lectin & alternative and only alternative pathway activation, a serially diluted serum sample (1∶1–1∶160) was applied to empty, human IgG, human IgA and properdin-coupled beads, respectively. The assay buffer for serum dilution contained either Ca^2+^-Mg^2+^, which promotes complement activation, or EDTA, which blocks complement activation. Complement activation-driven C3 fragment deposition on beads was detected by PE-conjugated anti-human C3 antibody. Plots display for each serum dilution the respective median fluorescence intensity (MFI) value against varying concentrations of human IgG, human IgA and properdin coupled on beads. AU - arbitrary units

Complement activation is an enzyme cascade that requires relatively higher serum concentrations than usually applied for antibody detection methods. For identifying the optimal serum dilution rate, human IgG, IgA or properdin were coupled on beads with the aim of initiating the activation of classical, lectin & alternative or only alternative pathways, respectively. Beads were incubated with serially diluted normal human serum and the level of complement activation was determined by detection of deposited C3 fragments using an anti-human C3–PE antibody. Complement activation on beads coupled to classical pathway activator human IgG decreased with the increased serum dilution rate, yet a low level of C3 was detected in 1∶160 diluted serum (***Figure 2B***). C3 detection for the beads coupled to human IgA, which is the lectin & alternative pathway activator, diminished when serum was diluted 1∶20 or more (***Figure 2C***). Complement activation on beads coupled to properdin, which stabilizes the C3 convertase enzyme of the alternative pathway and induces an extreme activation of the alternative pathway, could be detected even in serum diluted 1∶80 (***Figure 2D***). Very low MFI values for C3 detection on beads that were incubated in complement blocking EDTA supplemented buffer indicated that only the complement activation and not the passive binding of C3 molecules was measured by this method (***Figure 2B, 2C, 2D***).

Serum dilutions of 1∶5 or less resulted in remarkably elevated background as revealed by the signal intensities for the “empty” beads, which were not coupled to any protein or peptide (***Figure 2A***). Therefore, serum dilution rates of less than 1∶10 were not suitable due to high background. On the other hand, the higher the serum dilution rate, the lower the C3 fragment deposition was on complement activator-coupled beads, highlighting the trade-off between low background and high MFI values for C3 detection. Serum diluted 1∶10 in the Ca^2+^- Mg^2+^ - buffer resulted in high C3 signal intensities on activator-coupled beads and yet gave low background on empty beads. Thus, a serum dilution rate of 1∶10 was selected for use in further experiments.

### Pronounced complement activation masks detectability of antibodies

**Figure 3:**
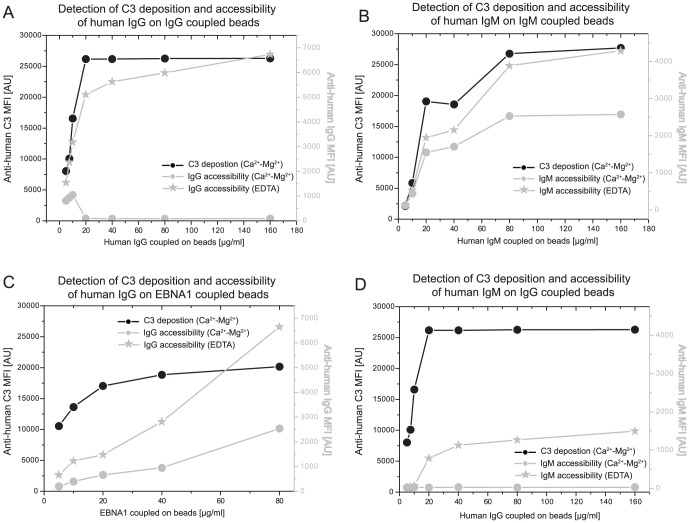
Complement activation masks detectability of antibodies. Human IgG *(* ***A,D*** *)*, human IgM *(* ***B*** *)* and EBNA-1 *(* ***C*** *)* coupled beads were incubated in 1∶10 diluted serum. Ca^2+^- Mg^2+^ (-•-) or EDTA (-*-) supplemented assay buffer was used for serum dilution. Anti-human C3-PE (black lines and axes), anti-human IgG-PE (gray lines and axes) or anti-human IgM-PE (gray lines and axes) secondary antibodies were used to measure the complement activation, IgG or IgM levels, respectively.

Antibody-antigen immune complexes or directly bead-bound human antibodies can activate the complement system. Complement activation results in the deposition of complement fragments on bead surface and this phenomenon can potentially mask the detection of bead-bound antibodies. In order to evaluate the masking effect of complement components on antibody detection, human IgG or IgM coupled beads were incubated with 1∶10 diluted normal human serum under assay conditions promoting (Ca^2+^- Mg^2+^ - buffer) or blocking (EDTA - buffer) complement activation. Directly bead-bound antibodies were detected by anti-human IgG – PE and anti-human IgM - Cy3 secondary antibodies. As ***Figure 3A and 3B*** show, substantial complement deposition entirely diminished the signal intensities for the anti-human IgG antibody and decreased the signal intensities for the anti-human IgM antibody. A remarkable loss of signal intensity for the anti-human IgG antibody was also observed when the viral antigen EBNA-1 coupled beads were incubated with a seropositive serum sample ***(*** ***Figure 3C*** ***)***. Furthermore, the masking effect of complement activation diminished detectability of IgG-specific IgM rheumatoid factors as well ***(*** ***Figure 3D*** ***)***. Based on these results, EDTA containing assay buffer was used for detection of bead bound serum antibodies.

### Detection of various rheumatoid factors and their complement activating properties

**Figure 4:**
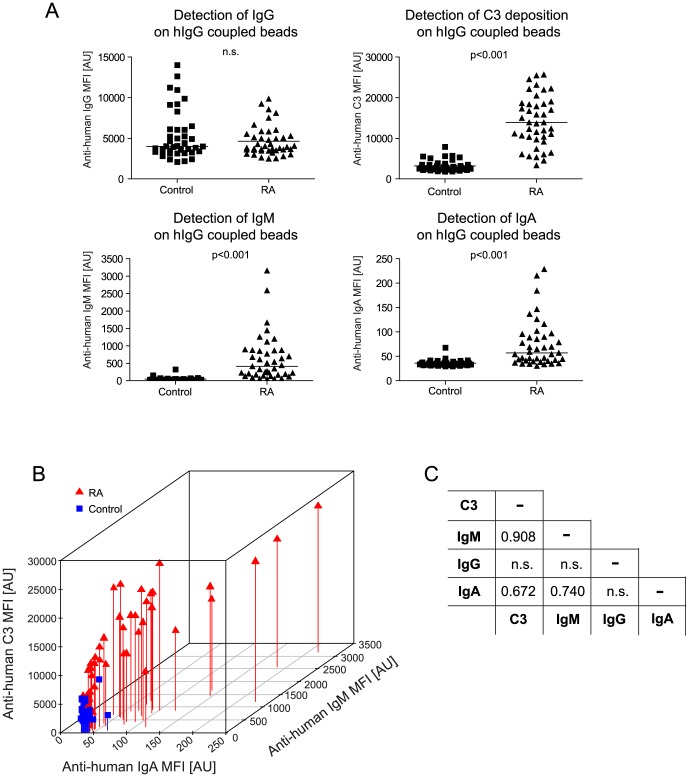
Complement activation by rheumatoid factors. Following incubation of human-IgG coupled beads in sera of RA patients or non-diseased controls, bound IgG-specific rheumatoid factors and deposited C3 fragments were detected. ***A*** *)* Median fluorescence intensities (MFI) for anti-IgG, anti-IgM, anti-IgA and anti-C3 secondary antibodies are plotted separately for the RA patients and controls. Statistical differences between these two groups were calculated by Mann-Whitney non-parametric test. ***B*** *)* Anti-IgA, anti-IgM and anti-C3 MFI values for the human-IgG coupled bead within RA patient group (Δ) and controls (▪) are plotted in a 3D graph for visualization of their correlation to each other. Anti-IgG MFI values are excluded from this plot since they not reveal a significant difference between the two groups. ***C)*** The table shows the Spearman's Rho correlation coefficients between anti-C3, anti-IgG, anti-IgM and anti-IgA MFI values in the entire sample cohort, where only statistically significant (p-value < 0.05) correlation coefficients are shown and non-significant correlations are indicated by (n.s.).

Sera of the majority of rheumatoid arthritis patients contain rheumatoid factors, namely autoantibodies against immunoglobulins [9]. Here, human IgG coupled beads were incubated with serum samples of RA patients and controls diluted 1∶10 in EDTA-containing assay buffer and the bound human antibodies were detected by anti-human IgG, anti-human IgM or anti-human IgA secondary antibodies ***(*** ***Figure 4*** ***)***. Anti-human IgG antibody expectedly recognized the human IgG molecules coupled on beads; thus there was no significant difference between RA patients and controls. On the other hand, RA serum samples revealed significantly higher MFI values than control samples for the anti-human IgM and anti-human IgA antibodies, demonstrating the presence of IgG-specific IgM and IgA rheumatoid factors in RA patient sera ***(*** ***Figure 4A*** ***, Figure S1)***. The level of rheumatoid factors of IgM isotype in some serum samples reached 3000 AU, which can be considered as a high value, since the maximum anti-IgM signal intensities for beads coupled with highest concentration of IgM revealed 7000 AU. The level of rheumatoid factors of IgA isotype was relatively low even in RA patients, since it reached only 250 AU where the maximum anti-IgA signal intensity for beads coupled with highest concentration of IgA revealed 6000 AU.

Levels of IgG-specific IgM rheumatoid factors were measured also by ELISA, which revealed a strong positive correlation (r = 0.927) between the MFI values obtained on the bead array platform and the OD values in ELISA ***(Figure S2A)***. In parallel, human IgG coupled beads were also incubated with serum samples diluted 1∶10 in Ca^2+^ and Mg^2+^-containing buffer, providing assay conditions promoting activation of the complement system and the level of C3 deposition on beads was measured by the anti-human C3 antibody. Complement activation on human IgG coupled beads was significantly higher in sera of RA patients compared to controls ***(*** ***Figure 4A*** ***).*** As ***Figure 4B and 4C*** show, there was a very strong positive correlation (r = 0.908) between the MFI values for anti-C3 and anti-IgM antibodies. The correlations between MFI values for anti-IgA and anti-IgM antibodies (r = 0.74) and anti-IgA and anti-C3 (r = 0.67) antibodies were comparable.

### Complement activating properties of autoantibodies against citrullinated peptides

Biotinylated citrulline or arginine-containing peptide pairs derived from filaggrin [36] and fibrinogen β [14] were coupled at their C- or N-terminal to neutravidin-coated beads through non-covalent but extremely strong and specific interaction, respectively. Serum samples needed first to be pre-adsorbed against neutravidin-specific antibodies before applying on bead arrays, since a number of serum samples were found to contain neutravidin-specific IgG and/or IgM antibodies, which would cause false positive signals ***(Figure S3A)***. Thus, both the Ca^2+^-Mg^2+^ or EDTA-containing assay buffers were supplemented with 100 µg/ml neutravidin and the serum samples were diluted 1∶10 in these buffers depending on whether C3 deposition or autoantibody level was measured.

**Figure 5:**
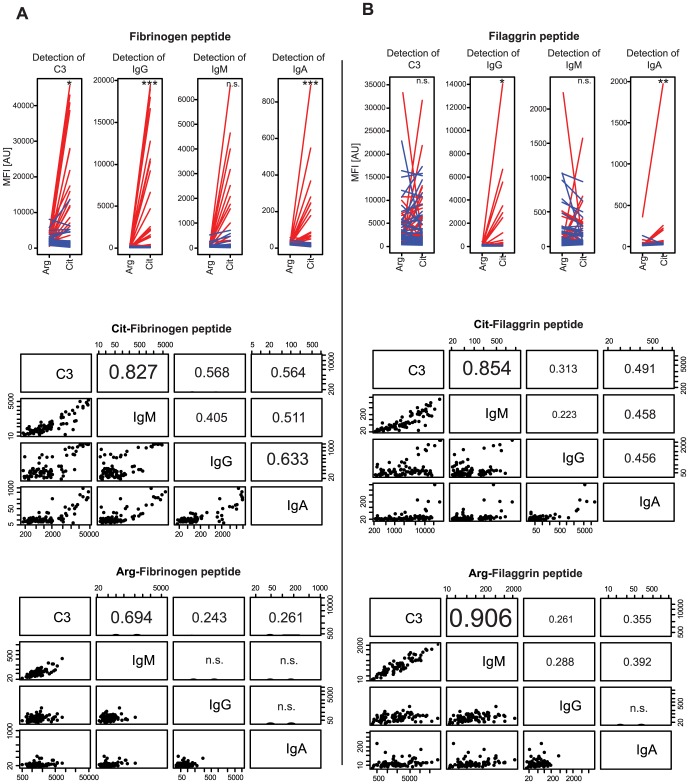
Detection of the level and the complement activating properties of autoantibodies against citrullinated peptides. The arginine or citrulline-containing form of fibrinogen β chain peptide (SGSG^60^RPAPPPISGGGYRAR^74^ vs. SGSG^60^XPAPPPISGGGYXAX^74^) (A) and filaggrin peptides (^454^TRGRS^458^K vs. ^454^TXGRS^458^K) (B) were coupled on beads and incubated with serum samples to detect peptide-specific IgG, IgM, IgA autoantibody levels and their complement activating properties. Median fluorescent intensity (MFI) values for arginine or citrulline-containing forms of the peptides in RA patient group (red lines) or the control group (blue lines) are shown in the upper panel. Significance level of differences between controls and RA patients were calculated only for the citrullinated peptides by Mann-Whitney test and the p-values are indicated in the upper panel as follow: * p-value<0.05; ** p-value<0.01, ***p-value<0.001. Spearman's Rho correlation coefficients calculated between anti-C3, anti-IgG, anti-IgM and anti-IgA MFI values are shown for citrullined (middle panels) or native (lower panels) form of the peptides, where non-significant correlations are indicated by (n.s.).

As ***Figure 5A*** shows, in sera of RA patients there were only minimal amount of autoantibodies of IgG, IgA or IgM isotypes against the arginine-containing form of fibrinogen β chain peptide (^60^RPAPPPISGGGYRAR^74^) in sharp contrast to its citrullinated form (^60^ **X**PAPPPISGGGY**X**A**X** ^74^). The autoantibodies against the citrullinated form of fibrinogen β peptide also induced an increased complement activation in the RA patient group. The results were different regarding the filaggrin-derived peptides: complement activating autoantibodies of IgM isotype against the arginine-containing form of filaggrin (^454^TRGRS^458^) were detected in sera of both RA patients and controls. On the other hand, citrullinated filaggrin peptide (^454^T**X**GRS^458^) specific IgG antibodies were detected only in sera of RA patients and not of controls. Peptide-specific IgG autoantibody levels in RA patient sera were also confirmed by ELISA, where significant correlations were revealed between bead array and ELISA measurements for the citrullinated forms of peptides for fibrinogen β and filaggrin ***(Figure S2B).***

### Antibodies against the viral antigen EBNA-1 and their complement activating properties

**Figure 6:**
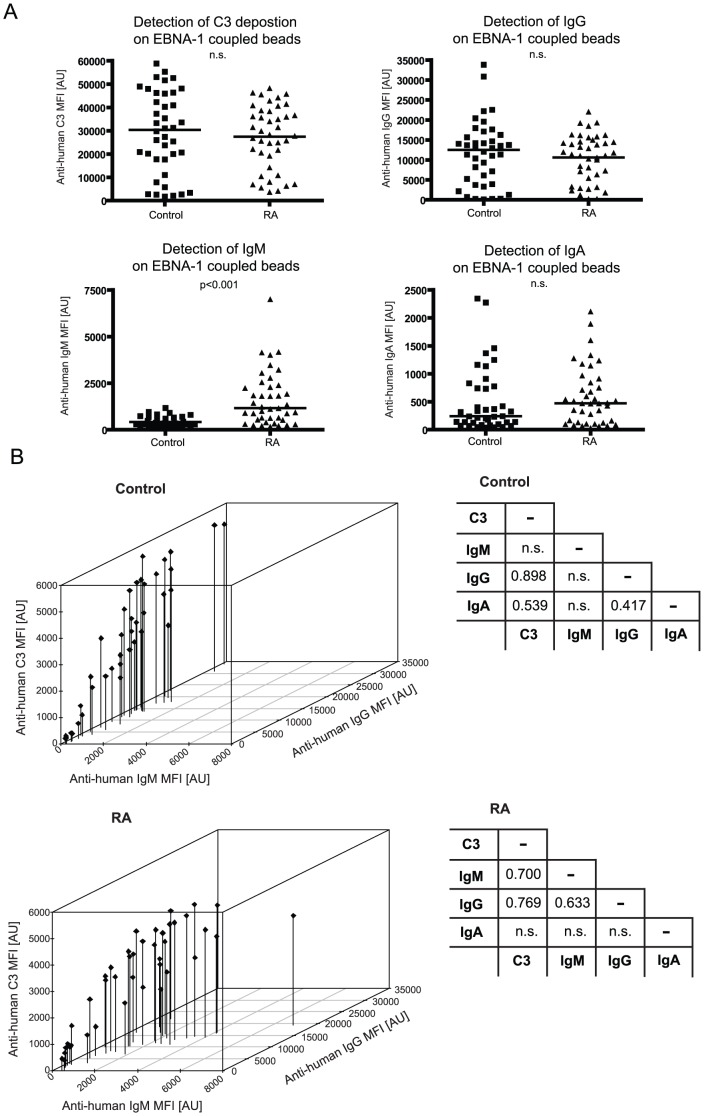
Level of EBNA-1 specific antibodies and their complement activation. EBNA-1 coupled beads were incubated with sera of RA patients and controls. ***A*** *)* Levels of bound human IgG, IgM and IgA antibodies along with the degree of complement activation are plotted for the two groups. Mann-Whitney non-parametric statistical test was used to calculate the statistical difference between the two groups. ***B*** *)* Signal intensities for anti-IgM, anti-IgA and anti-C3 for the controls (upper panel) and RA patients (lower panel) are plotted in a 3D graph. The tables show the Spearman's Rho correlation coefficients between anti-C3, anti-IgG, anti-IgM and anti-IgA signal intensities calculated separately for the control and RA patient groups. Only statistically significant (p-value<0.05) correlation coefficients are shown and non-significant correlations are indicated by (n.s.).

EBNA-1-specific IgG, IgM, IgA levels and their complement activating properties were measured in serum samples of RA patients and controls. Only the MFI values for the anti-IgM antibody on EBNA-1 coupled beads were significantly higher in sera of RA patients compared to controls ***(*** ***Figure 6A*** ***)***. There was also a significantly strong (r = 0.714) positive correlation between the levels of IgG-specific IgM rheumatoid factors and the anti-IgM signal intensities for EBNA-1 coupled beads ***(Figure S4.)*** Since most of the serum samples contain EBNA-1 specific IgG, the discriminating property of IgM signal intensities for EBNA-1 was supposedly derived from the rheumatoid factors that would bind to the EBNA-1 specific IgG antibody. Accordingly, there was a very high (r = 0.898) correlation between EBNA-1-specific C3 and IgG levels especially in control serum samples, which are not affected by the interference of rheumatoid factors ***(*** ***Figure 6B*** ***)***. The correlation between EBNA-1-specific IgA and C3 deposition was lower (p = 0.539) in control serum and non-significant in RA samples.

## Discussion

We herein describe the development and application of a suspension-bead array based workflow for multiplex and parallel measurement of antigen-specific antibodies and complement activation in sera. To our current knowledge, this is the first study demonstrating the use of multiplex bead arrays for parallel measurement of antibody reactivity and complement activation towards various peptides and viral proteins in clinical samples within the same assay.

The complement system is an important part of the immune system and assessing the degree of complement activation can reveal important information about various pathophysiological processes. Human antibodies of different isotypes in complex with an antigen can induce complement activation to different extents. In general, the property of human antibody isotypes regarding activation of the classical pathway is as follows: IgM, IgG3>IgG1>>IgG2, while IgG4 and IgE do not activate the complement system and IgA activates only the alternative and lectin pathways [25]. Yet, different antibody clones of same isotype can initiate complement activation with very different efficacy [26], thus the direct measurement of antigen-specific complement activation has many advantages. The clinical practice currently mainly focuses on measurement of overall complement activation and determines CH50 value or the level of C3 and C4 in the serum. ELISA tests allowing a separate analysis of the three complement activation pathways are also available [27], [28]. However, when the assessment of antigen-specific complement activation is the goal, especially with the need to evaluate several candidate antigens in parallel, highly-multiplex systems such as planar [29] or suspension bead arrays offer a great advantage.

Wahrmann *et al.* previously showed a flow cytometry-based bead system to measure HLA alloantigen induced complement activation. They suggest detection of deposited C1q or C4 split products as an indication of complement activation and their data argues against the use of C3 fragment measurement, since it resulted in an alloantibody independent high background [30]. The authors applied undiluted serum that resulted in a very high background in our assay as well, but as ***Figure 2*** shows, if the bead array was incubated with a 1∶10 diluted serum the background for the C3 detection disappeared and the antibody-dependent complement activation was measureable. In other published studies, C4 deposition was measured using an undiluted external complement source [31]–[34]; or bound externally added purified human C1q was detected after the antigen-coupled beads were treated with a mixture of heat-inactivated serum sample and C1q [35]. We measured C3 deposition as this reflects activation of any of the three pathways, which is particularly favorable since ACPAs can induce complement activation both through the classical and alternative pathways [36].

The own complement system of the tested serum sample itself was not utilized in any of these previous studies. Yet, utilizing the serum sample's own complement system can be favorable, since it reflects more precisely the individual-specific processes in the investigated serum sample as not only the presence of the complement activating antibody but also a responsive complement system is necessary for the complement activation. Avoiding the use of an external complement source can be especially attractive when highly multiplexed systems are used. External complement sources mostly consist of a mixture of normal human sera and the more ligand are tested the higher the possibility of getting false positive results due to using this serum mixture. On the other hand, inappropriate sample preparation, storage and transportation conditions or disease-related systemic complement consumption can decrease the complement activating capability of patient samples, which might lead to false negative results when the sample's own complement system is used as the complement source. Yet, in the presented assay setup, the intactness of the complement system in each sample can be easily inspected by including properdin-coupled beads in the assay. Properdin induces an extreme activation of the alternative pathway leading to C3 fragment deposition [37] and is more suitable as an internal control than the human IgG, as the RF would not alter the amount of bound C3 fragments. Very low amount of C3 deposition on properdin coupled beads indicates decreased complement level and necessitates sample exclusion or further normalization. By demonstrating that the complement system in the tested serum samples was intact and functioning with similar efficiency (***Figure S5***), we have used the own complement system of the serum samples which required a very strictly controlled storage of serum sample aliquots at −70°C by avoiding any repeated freeze-thaw cycles.

Here, an assay buffer containing physiologically equivalent concentrations of Ca^2+^ and Mg^2+^ ions was used for dilution of serum samples since these ions are essential for the activation of classical, lectin or alternative pathways, respectively. In addition, a relatively more concentrated serum condition is needed for measurement of complement activation than usually applied for detection of antibodies, where generally 1∶100 or even more diluted serum can be utilized. Harboe *et al.* reported that hemolytic activity of the serum is lost at 1∶16 dilution for assaying the alternative pathway and at 1∶1024 dilution for assaying the classical pathway [38]. We have previously shown that 1∶5–1∶10 serum dilutions give the highest signal-to-noise ratios for complement measurement on nitrocellulose-coated protein microarray slides [39]. Trouw *et al.* also used a serum dilution of 1∶10 in their ELISA based ACPA induced complement activation measurements [36]. Optimization of the serum dilution rate is though necessary for each assay platform, as the level of complement activation depends also on the type of the surface coating.. We observed a very high background on the empty beads when applying less than 1∶5 times diluted serum, which is presumably the consequence of continuous alternative pathway tick-over that gives rise to reactive C3 fragments ***(*** ***Figure 2*** ***)***. Yet, serum diluted 1∶10 in the presence of physiologically equivalent concentrations of Ca^2+^ and Mg^2+^ ions provided optimal conditions for complement measurement. But as ***Figure 3*** shows, this condition was not ideal for a parallel measurement of bound antibodies. Extensive complement fragment deposition on the site of activation masks the bound antibodies and prevents their detectability by the secondary antibodies. This effect was more pronounced regarding the detection of IgG than IgM ***(*** ***Figure 3*** ***)***. To circumvent this effect, either higher serum dilutions can be utilized [39] or the assay buffer can be supplemented with EDTA, which blocks the complement activation. Here we used the latter approach since our aim was to measure antibody levels under assay conditions as identical as possible to the conditions used for measurement of complement activation.

Here we have demonstrated the utility of multiplex antigen-specific complement activation measurement on the bead array platform both in the context of an autoimmune disease (rheumatoid arthritis) and for detection of a viral antigen (EBNA-1). There are evidences that complement activation contributes to the pathological processes of rheumatoid arthritis [40]. Rheumatoid factors are anti-human IgG Fc-specific autoantibodies of various isotypes (IgM, IgG, IgA, IgE) [41] and some of these autoantibodies can activate the complement system. There are discrepancies among the findings of previous studies concerning the prognostic value of rheumatoid factor isotypes [10], [42]. We determined the levels of rheumatoid factors in sera of RA patients and non-diseased controls using human IgG coupled beads ***(*** ***Figure 4*** ***)***. The serum samples in the RA patient group revealed a significantly higher level of rheumatoid factors of IgM and IgA isotype than the control group, which is in agreement with the findings of previous studies [10]. Rheumatoid factors especially of IgM isotype induced very strong complement activation and C3 fragment deposition. The correlation between the abundance of rheumatoid factors of IgM isotype and C3 deposition was very strong (r = 0.908) ***(*** ***Figure 4C*** ***)*** and the classification power of IgM rheumatoid factors between RA patients and controls was high (AUC>0.97) ***(Figure S1)***. Samples collected from patients with a disease other than RA were not included in this study, as it would go beyond the scope of this study. Though in such a case, the classification power of rheumatoid factors might presumably be lower as high levels of rheumatoid factors are known to be present also in other diseases [11].

Measurement of anti-citrullinated peptide antibodies (ACPAs) in the context of RA shows higher specificity; in this study two peptide epitopes, one from fibrinogen β chain [14] and another one from filaggrin [9], [22] protein were tested. These peptides represented the most dominant epitopes and were investigated in more detail ***(*** ***Figure 5*** ***)***. IgG reactivity against the citrullinated version of both fibrinogen β and filaggrin peptides were exclusively observed only in the sera of RA patients and not in controls. IgA reactivity against the citrullinated fibrinogen β peptide and C3 deposition also significantly discriminated RA patients from the controls. On the other hand, there were no significant differences between RA patients and controls regarding the IgM reactivity against both of the peptides. Especially in case of the filaggrin peptide, IgM autoantibodies recognizing even the native form of the peptide were present in sera of both RA patients and controls. These findings are in agreement with the results of our previous study, where the same filaggrin peptide pair was utilized in a planar protein microarray system [43]. As revealed by the strong correlation between anti-IgM and anti-C3 signal intensities (r>0.8), the IgM autoantibodies against the filaggrin peptide present in sera of both sample cohorts could activate the complement system and did not result in a significant difference between RA patients and controls regarding the C3 levels.

Pratesi *et al.* suggested that also citrullinated forms of viral proteins such as the Epstein-Barr virus nuclear antigen 1 can be targets of ACPAs [16]. However, we utilized here only the native form of the EBNA-1 recombinant protein as a representative viral antigen and not because of any potential relevance to RA. Based on the fact that the majority of the population worldwide is seropositive for EBV, antibodies in sera against the immunodominant EBNA-1 protein are widespread [44] and we have confirmed this in our study as well ***(*** ***Figure 6A*** ***)***. Yet, we observed that EBNA-1 specific IgM reactivity significantly differed between RA patients and the controls. To elucidate this observation further, we investigated the correlation between the IgM reactivity against EBNA-1 and the level of rheumatoid factors of IgM isotype, which indeed revealed a strong correlation ***(Figure S4).*** Thus, the difference between RA patients and controls regarding the IgM reactivity against EBNA-1 is presumably caused as a pitfall by the presence of rheumatoid factors of IgM isotype in RA patient sera. Also Henle *et al.* previously found that rheumatoid factors can cause false-positive results in tests for EBNA-1 specific IgM antibodies [45]. The high level of complement-activating EBNA-1 specific IgG antibodies presumably overrode the complement activation of rheumatoid factors since the C3 signal intensities for EBNA-1 was high in sera of both the RA patients and controls. The interfering effect of rheumatoid factors regarding the EBNA-1 reactivity dictated to separately investigate the data derived from the RA patients and controls. In the control group, where the interfering effect of rheumatoid factors was not present, there was a very strong correlation (r = 0.898) between complement activation and IgG level ***(*** ***Figure 6B*** ***)***. IgA reactivity against EBNA-1 in a portion of the individuals was present in both sample groups. EBNA-1 protein contains a glycil-alanine (Gly-Ala) repeat region that was found as a dominant IgA antigen epitope for instance in patients with nasopharyngeal carcinoma but it was also shown that non-diseased serum samples might represent IgA reactivity against it, although with lower prevalence [46].

In conclusion, the findings we present here illustrate that antigen-specific immunoglobulin reactivity of various isotypes towards autoantigens or viral antigens and antigen-specific complement activation can be measured within the same assay and in a multiplex format using the suspension bead array platform. Measuring the C3 deposition allowed here to assess the joint effect of the three different possible complement pathways. Here we detected auto- and viral antigen-specific antibodies in the sera of RA patients and non-diseased controls, we investigated their complement activating properties and we also showed that rheumatoid factors have an interfering effect on complement activation measurement potentially causing false positive results. The suspension bead array-based workflow we developed and implemented here can be utilized to investigate any other autoimmune or infectious disease where the presence of complement activating antibodies has diagnostic value. Therefore, the presented approach is an important advancement for the analysis of autoantibody- or virus-associated immune pathologies.

## Supporting Information

Figure S1 **Classification power of rheumatoid factors and their complement activating properties.** C3 fragment deposition and binding of IgM, IgG, IgA antibodies were measured on human-IgG coupled beads incubated with sera of 40 non-diseased controls and 41 RA patients. Receiver operating characteristic (ROC) curves and area under the curve (AUC) values are displayed on the figure.
(PDF)

Figure S2 **Correlation between the bead array and ELISA measurements.** ***A*** *)* Level of human IgG-specific IgM was determined in sera of RA patients by ELISA and on the bead array platform. Here, an ELISA plate was coated overnight with 5 µg/ml of human IgG in 0.05 M carbonate buffer, pH 9.5. Following washing with PBS-Tween, wells were blocked with blocking buffer (1% BSA, 0.05% Tween in PBS) at 37°C for 30 min. Wells were incubated in 200x diluted serum sample (diluted in blocking buffer) at 37°C for 1h. Following washing, bound human IgM was detected by rabbit anti-human IgM-HRP conjugate at 37°C for 1h. After TMB substrate development, OD was measured at 450 nm (reference 620 nm). ***B*** *)* Peptide-specific IgG levels in sera of RA patients and controls were also measured both by ELISA and on the bead array platform. For ELISA measurements, biotinylated peptides (1 µg/ml in PBS) were bound to neutravidin (5 µg/ml in PBS) pre-coated plates. Following washing with PBS-Tween, plates were blocked with blocking buffer (150 mM NaCl, 2% BSA in PBS) at 37°C for 30 min, then serum samples were added (1∶100) in dilution buffer (2 M NaCl, 2% BSA in PBS). After an overnight incubation and washing the plate, rabbit anti-human IgG-HRP conjugate was added for 1h. Following TMB substrate development, OD was measured at 450 nm (reference 620 nm). The values derived from the ELISA and the bead array platform are plotted and Spearman' Rho correlation coefficients are indicated. OD - optical density; ELISA - enzyme-linked immunosorbent assay.
(PDF)

Figure S3 **Presence of and pre-adsorption against neutravidin-specific antibodies in sera.** ***A*** *)* Protein microarray technique was applied to determine the neutravidin-specific IgG and IgM levels in sera of each of the controls and RA patients. In short, 0.33 mg/ml neutravidin was printed in triplicates onto nitrocellulose covered glass slides by BioOdyssey Calligrapher miniarrayer (Bio-Rad). Following washing steps with PBS, slides were incubated with 1∶20 diluted serum sample (25 mM EDTA, 5% BSA, 0.05% Tween 20 in PBS) at 37°C for 1h. Bound antibodies were detected by 1∶2500 diluted DyLight 488-conjugated F(ab')_2_ fragment of goat anti-human IgM (µ chain specific) and DyLight 649-conjugated F(ab')_2_ fragment of goat anti-human IgG, (γ chain specific) (Jackson ImmunoResearch) antibodies. The neutravidin-specific IgG and IgM fluorescence intensity (FI) values of each serum sample are plotted. The arrow indicates the selected serum sample that was used in further experiments on the bead array platform. ***B*** *)* Neutravidin or EBNA-1 coupled beads were incubated in 1∶10 diluted, untreated (black) or neutravidin pre-adsorbed (gray) serum. Here, serum was diluted in Ca^2+^-Mg^2+^ - supplemented buffer for C3 detection and in EDTA-supplemented buffer for IgG and IgM detection. Neutravidin pre-adsorption diminished neutravidin-specific C3 and IgG levels, while it substantially decreased IgM levels. Neutravidin pre-adsorption had no effect on EBNA-1-specific signal intensities. ***C*** *)* Furthermore, neutravidin pre-adsorption had no effect on overall complement activation: classical pathway activator human IgG and alternative pathway activator properdin were coupled on beads at varying concentrations and incubated with 1∶10 diluted, untreated (black square) or neutravidin pre-adsorbed (gray circle) serum. C3 fragment deposition was detected by the anti-human C3-PE antibody and the plots display the resulting MFI values.
(PDF)

Figure S4 **Correlation between anti-IgM signal intensities for the viral antigen EBNA-1 and for human IgG-coupled beads.** Anti-IgM MFI values for EBNA-1-coupled beads and for human IgG- coupled beads are plotted and Spearman's Rho correlation coefficient is indicated.
(PDF)

Figure S5 **Distribution of anti-C3 signal intensities for properdin-coupled beads.** Anti-C3 MFI values for all the tested serum samples on properdin-coupled beads are plotted. MFI values for all the samples were within 3 standard deviations (± 3SD) of the median.
(PDF)
