# Supplementary figures and images for: Bead Arrays for Antibody and Complement Profiling Reveal Joint Contribution of Antibody Isotypes to C3 Deposition
Source: PLoS One. 2014 May 5;9(5):e96403. doi: 10.1371/journal.pone.0096403 (PMC4010547; doi:10.1371/journal.pone.0096403)

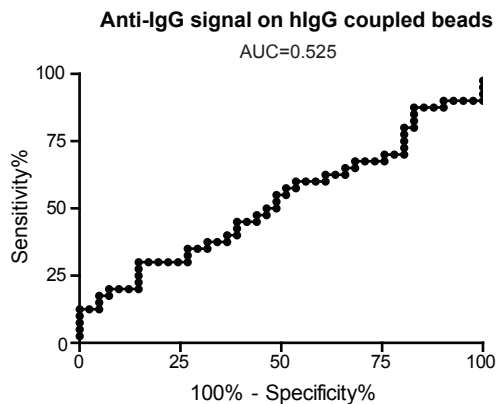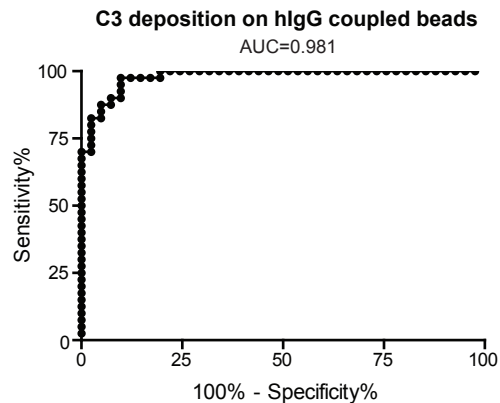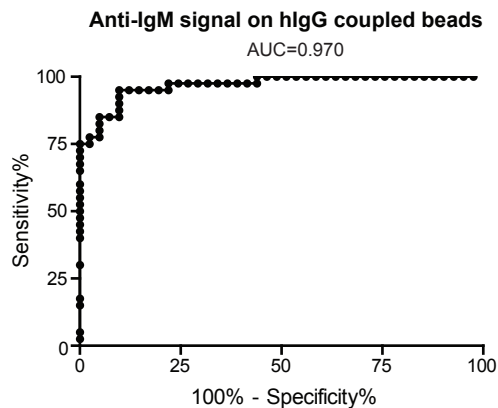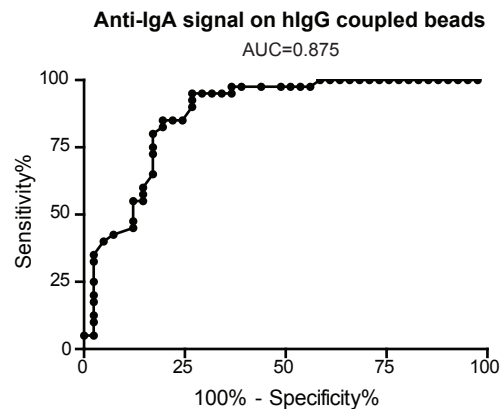

**Figure S1.**

Supplement: Figure S1 — Classification power of rheumatoid factors and their complement activating properties. C3 fragment deposition and binding of IgM, IgG, IgA antibodies were measured on human-IgG coupled beads incubated with sera of 40 non-diseased controls and 41 RA patients. Receiver operating characteristic (ROC) curves and area under the curve (AUC) values are displayed on the figure. (PDF) [file pone.0096403.s001.pdf]

**A**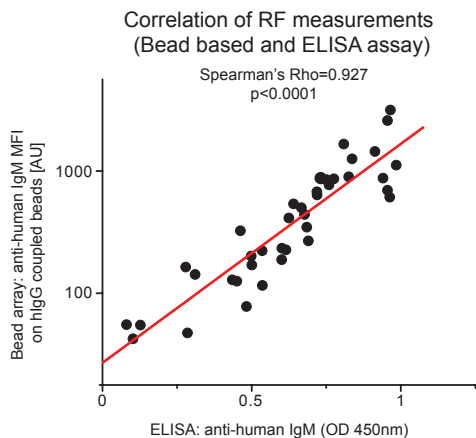**B**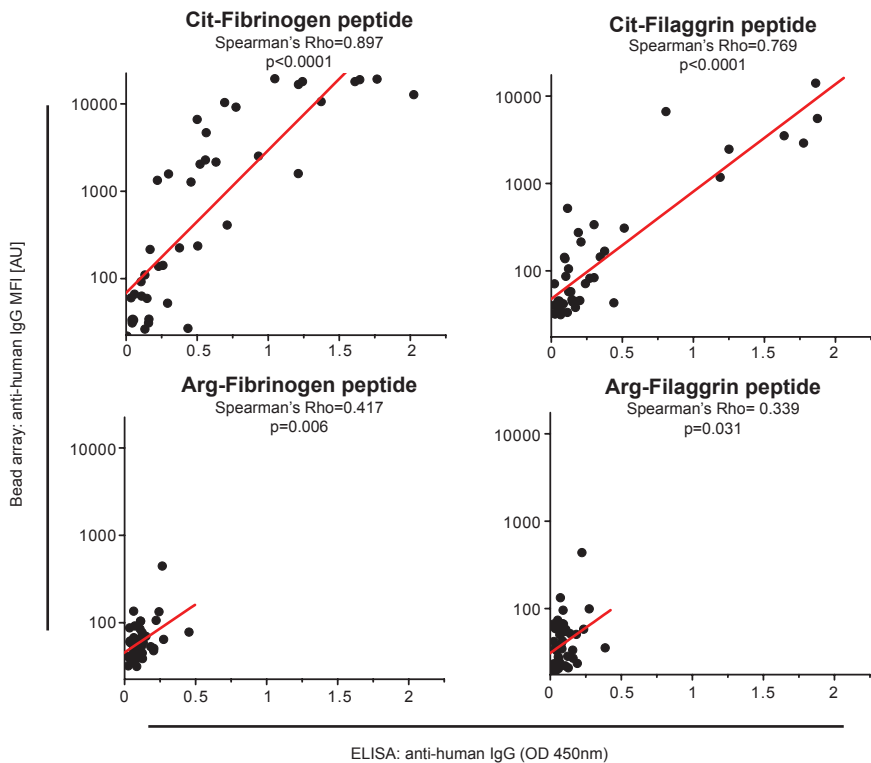**Figure S2.**

Supplement: Figure S2 — Correlation between the bead array and ELISA measurements. A ) Level of human IgG-specific IgM was determined in sera of RA patients by ELISA and on the bead array platform. Here, an ELISA plate was coated overnight with 5 µg/ml of human IgG in 0.05 M carbonate buffer, pH 9.5. Following washing with PBS-Tween, wells were blocked with blocking buffer (1% BSA, 0.05% Tween in PBS) at 37°C for 30 min. Wells were incubated in 200x diluted serum sample (diluted in blocking buffer) at 37°C for 1h. Following washing, bound human IgM was detected by rabbit anti-human IgM-HRP conjugate at 37°C for 1h. After TMB substrate development, OD was measured at 450 nm (reference 620 nm). B ) Peptide-specific IgG levels in sera of RA patients and controls were also measured both by ELISA and on the bead array platform. For ELISA measurements, biotinylated peptides (1 µg/ml in PBS) were bound to neutravidin (5 µg/ml in PBS) pre-coated plates. Following washing with PBS-Tween, plates were blocked with blocking buffer (150 mM NaCl, 2% BSA in PBS) at 37°C for 30 min, then serum samples were added (1∶100) in dilution buffer (2 M NaCl, 2% BSA in PBS). After an overnight incubation and washing the plate, rabbit anti-human IgG-HRP conjugate was added for 1h. Following TMB substrate development, OD was measured at 450 nm (reference 620 nm). The values derived from the ELISA and the bead array platform are plotted and Spearman' Rho correlation coefficients are indicated. OD - optical density; ELISA - enzyme-linked immunosorbent assay. (PDF) [file pone.0096403.s002.pdf]

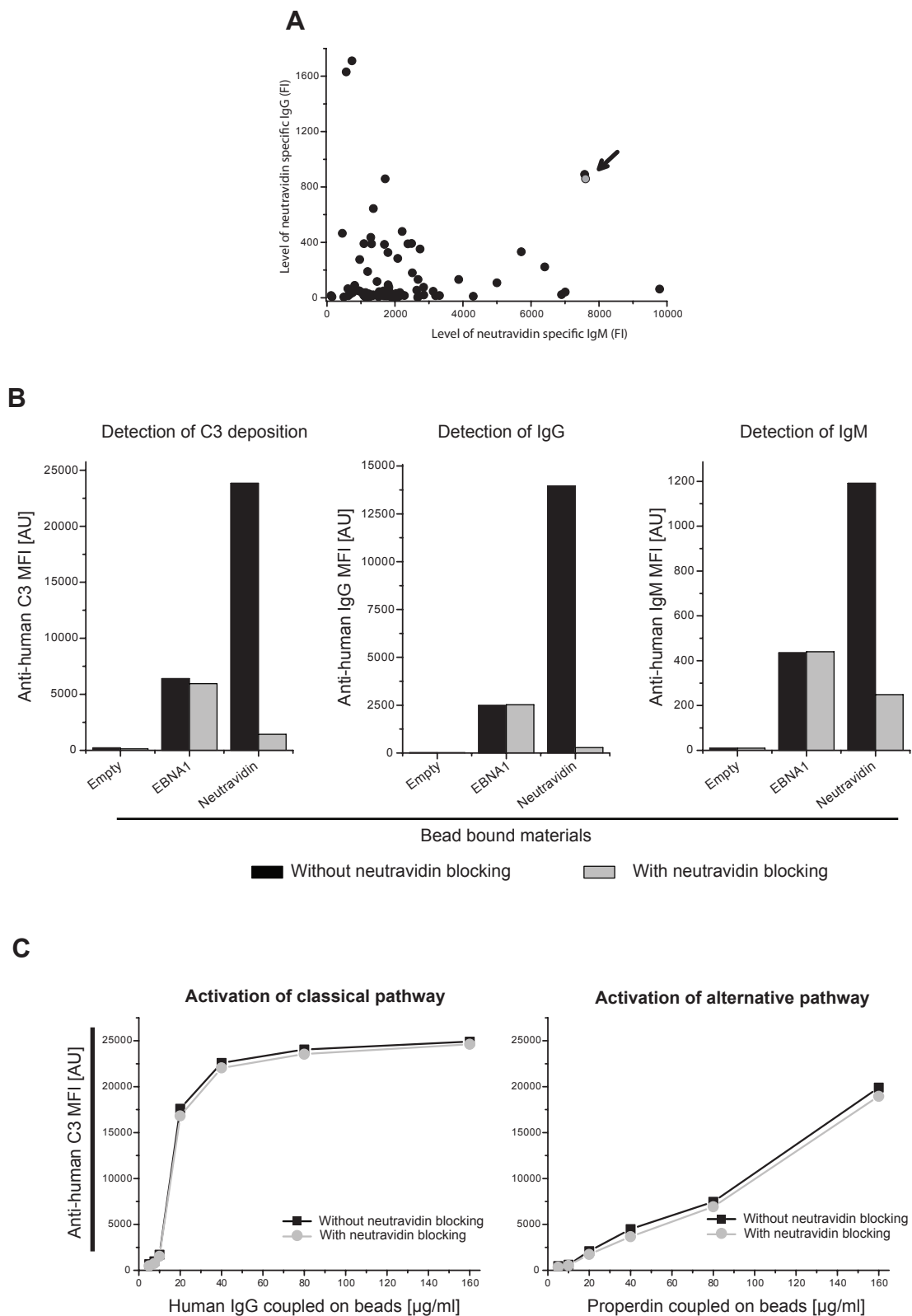

**Figure S3**

Supplement: Figure S3 — Presence of and pre-adsorption against neutravidin-specific antibodies in sera. A ) Protein microarray technique was applied to determine the neutravidin-specific IgG and IgM levels in sera of each of the controls and RA patients. In short, 0.33 mg/ml neutravidin was printed in triplicates onto nitrocellulose covered glass slides by BioOdyssey Calligrapher miniarrayer (Bio-Rad). Following washing steps with PBS, slides were incubated with 1∶20 diluted serum sample (25 mM EDTA, 5% BSA, 0.05% Tween 20 in PBS) at 37°C for 1h. Bound antibodies were detected by 1∶2500 diluted DyLight 488-conjugated F(ab')2 fragment of goat anti-human IgM (µ chain specific) and DyLight 649-conjugated F(ab')2 fragment of goat anti-human IgG, (γ chain specific) (Jackson ImmunoResearch) antibodies. The neutravidin-specific IgG and IgM fluorescence intensity (FI) values of each serum sample are plotted. The arrow indicates the selected serum sample that was used in further experiments on the bead array platform. B ) Neutravidin or EBNA-1 coupled beads were incubated in 1∶10 diluted, untreated (black) or neutravidin pre-adsorbed (gray) serum. Here, serum was diluted in Ca2+-Mg2+ - supplemented buffer for C3 detection and in EDTA-supplemented buffer for IgG and IgM detection. Neutravidin pre-adsorption diminished neutravidin-specific C3 and IgG levels, while it substantially decreased IgM levels. Neutravidin pre-adsorption had no effect on EBNA-1-specific signal intensities. C ) Furthermore, neutravidin pre-adsorption had no effect on overall complement activation: classical pathway activator human IgG and alternative pathway activator properdin were coupled on beads at varying concentrations and incubated with 1∶10 diluted, untreated (black square) or neutravidin pre-adsorbed (gray circle) serum. C3 fragment deposition was detected by the anti-human C3-PE antibody and the plots display the resulting MFI values. (PDF) [file pone.0096403.s003.pdf]

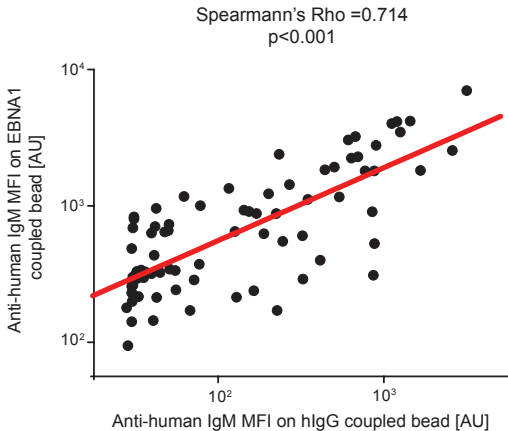

**Figure S4.**

Supplement: Figure S4 — Correlation between anti-IgM signal intensities for the viral antigen EBNA-1 and for human IgG-coupled beads. Anti-IgM MFI values for EBNA-1-coupled beads and for human IgG- coupled beads are plotted and Spearman's Rho correlation coefficient is indicated. (PDF) [file pone.0096403.s004.pdf]

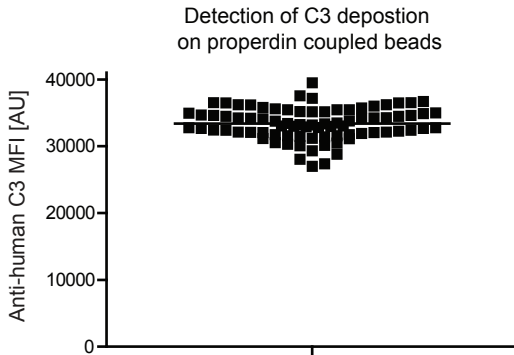

**Supplementary Figure 5.**

Supplement: Figure S5 — Distribution of anti-C3 signal intensities for properdin-coupled beads. Anti-C3 MFI values for all the tested serum samples on properdin-coupled beads are plotted. MFI values for all the samples were within 3 standard deviations (± 3SD) of the median. (PDF) [file pone.0096403.s005.pdf]
